# Revealing the queer-spectrum in STEM through robust demographic data collection in undergraduate engineering and computer science courses at four institutions

**DOI:** 10.1371/journal.pone.0264267

**Published:** 2022-03-10

**Authors:** A. M. Aramati Casper, Rebecca A. Atadero, Linda C. Fuselier

**Affiliations:** 1 Civil and Environmental Engineering Department, Colorado State University, Fort Collins, Colorado; 2 Graduate Degree Program in Ecology, Colorado State University, Fort Collins, Colorado; 3 Department of Biology, Colorado State University, Fort Collins, Colorado; 4 Biology Department, University of Louisville, Louisville, Kentucky; University of Texas Southwestern Medical Center at Dallas, UNITED STATES

## Abstract

Queer identities are often ignored in diversity initiatives, yet there is a growing body of research that describes notable heterosexist and gender-normative expectations in STEM that lead to unsupportive and discriminatory environments and to the lower persistence of queer individuals. Research on the experiences of queer-spectrum individuals is limited by current demographic practices. In surveys that are queer-inclusive there is no consensus on best practices, and individuals with queer genders and queer sexual, romantic, and related orientations are often lumped together in a general category (e.g. LGBTQ+). We developed two queer-inclusive demographics questions and administered them as part of a larger study in undergraduate engineering and computer science classes (n = 3698), to determine which of three survey types for gender (conventional, queered, open-ended) provided the most robust data and compared responses to national data to determine if students with queer genders and/or queer sexual, romantic, and related orientations were underrepresented in engineering and computer science programs. The gender survey with queer-identity options provided the most robust data, as measured by higher response rates and relatively high rates of disclosing queer identities. The conventional survey (male, female, other) had significantly fewer students disclose queer identities, and the open-ended survey had a significantly higher non-response rate. Allowing for multiple responses on the survey was important: 78% of those with queer gender identities and 9% of those with queer sexual, romantic and related orientations selected multiple identities within the same survey question. Queer students in our study were underrepresented relative to national data. Students who disclosed queer gender identities were 7/100ths of the expected number, and those with queer orientations were under-represented by one-quarter. Further work developing a research-based queered demographics instrument is needed for larger-scale changes in demographics practices, which will help others identify and address barriers that queer-spectrum individuals face in STEM.

## Introduction

The heterosexist and gender-normative biases in STEM fields have the potential to create unsupportive environments for queer-spectrum individuals (i.e. not cisgender and/or not heterosexual) [1–7]. Specifically, queer-spectrum STEM professionals and students experience exclusion from networking and resources, harassment, devaluating of their contributions, a more negative work environment, decreased professional success, and a chilly climate towards any discussion of their identity, as queer identities are often seen as irrelevant and not to be discussed [1–3, 8–13]. The consequences of this chilly climate include queer-spectrum students’ under-representation and lower persistence than their cisgender and heterosexual peers, and the higher likelihood of queer-spectrum STEM professionals to consider and create plans to leave not only their profession, but STEM completely [1, 7, 14–16]. Demographic collection practices often exacerbate this chilly climate, as surveys often have limited gender options and rarely ask questions relating to sexual, romantic, and related orientations [1, 17–20], limiting research on the experiences of queer-spectrum people [21–24].

One challenge in identifying and addressing the challenges that queer-spectrum individuals experience is the continually evolving terminology around gender and sexual, romantic, and related identities. We drew from language used by related research in STEM spaces that use the term queer as an umbrella term [e.g. 25–27], while also differentiating between gender identities and sexual, romantic, and related orientations. Therefore, we use the term *queer-spectrum* as an umbrella term for all those with non-cisgender and/or non-heterosexual identities. Under that umbrella term, we use *queer gender* to encompass all non-cisgender genders, and *queer sexual*, *romantic*, *and related orientations* to reference all non-heterosexual orientations. While we realize that not all individuals within a given umbrella term will necessarily identify with that term, this is true of any terminology that is used to define groups of people with queer-spectrum identities [22, 27].

Data collection is a key step toward understanding successes and failures of educating and supporting queer-spectrum people in STEM [7, 16, 28]. The importance of collecting data on the experiences of queer-spectrum people is exemplified by how participation in undergraduate research experiences, a strong predictor of STEM retention overall, does not predict the persistence of queer-spectrum students [16, 29], as well as the widespread inequities for queer-spectrum STEM professionals revealed in Cech and Waidzunas’ recently published large-scale survey data [1]. However, even in surveys that create space for queer-spectrum identities, there is no consensus on best practices, leading to a range of data collection practices that may or may not accurately capture individuals’ identities and that yield data that are not necessarily comparable [21–24, 28, 30, 31].

Although collecting reliable data on queer-spectrum individuals can be difficult because of inconsistencies in defining identities, counting and quantifying queer-spectrum individuals is vital for awareness and action toward societal change [22, 32]. Demographic data are important in justifying funding for services that work to change the current exclusionary environment, within and beyond STEM fields [22]. Yet, many population-level surveys do not include queer-spectrum demographic options. Two recent nation-wide surveys begin to fill this previous void, a survey performed by Harris Poll for GLADD in 2017 [33] and a Gallup Poll in 2020 [34]. The Harris Poll found that overall 20% of 18–35 year olds identified as LGBTQ and the Gallup Poll found that 16% of generation z (born from 1997–2002), identified as LGBT. These two polls collected data differently, with the Gallup Poll specifically focusing on lesbian, gay, bisexual, and transgender (LGBT) identities, and thus a narrower scope of identities and therefore people, whereas the Harris Poll was framed much more broadly, explicitly including asexual, pansexual, queer, questioning, agender, genderfluid, bigender, and genderqueer identities. Despite these differences in scope, both indicate that younger people are much more likely to identify on the queer spectrum. Additionally, the Harris Poll found that younger participants were much more likely to identify with identities beyond those included in narrower LGBT categories.

Importantly, both surveys demonstrate that if the percentage of queer-spectrum students in STEM classes was representative, roughly 1 in 5 to 1 in 7 students in a given class would be likely to have a queer-spectrum identity. And therefore, to create a more inclusive STEM environment, broadly collected demographic data need to be queer-spectrum inclusive so the data can be disaggregated at the sub-population level and used to differentiate and contextualize the experiences of queer individuals [35]. Our study is one of the first to a) analyze the disaggregated representation of students with queer gender identities and queer sexual, romantic, and related orientations in engineering and computer science undergraduate degree programs, and b) specifically explore how best to design questions to collect disaggregated demographic data on queer-spectrum students in STEM disciplines by separating gender identities from sexual, romantic, and related orientations. The purposes of this paper are to: 1) determine if queer students are underrepresented in engineering and computer science classes; 2) provide recommendations for demographic question formats based on students’ responses to three different survey types; and 3) ultimately, enhance queer-spectrum inclusive demographic data collection practices that disaggregate gender identity and sexual, romantic, and related orientations.

### Challenges and key considerations for collecting queer-inclusive data

Shifting demographic data collection practices to include queer-spectrum individuals has unique challenges, including: 1) developing options that reflect current terminology and that are also geographically and culturally relevant, and 2) existing risks to individuals completing the survey, as queer-spectrum identities are not well protected. Additionally, researchers collecting these data may experience negative consequences within their institutions or disciplines.

#### Relevant language

Language used by queer-spectrum individuals to describe their identity(ies) is rapidly shifting and diversifying and can vary both regionally and across generations [33, 35, 36]. For example, while “transgendered” was previously acceptable, it is now considered an outdated and problematic term because, as a verb, it implies that something happens to someone rather than describing an identity. Instead, the term “transgender,” which is an adjective, is now the appropriate language to use. Furthermore, unless one’s transgender identity is particularly relevant in a given situation, it is most appropriate to simply refer to someone as the gender they identify as (e.g. “woman” or “man”), instead of calling them out as a “transgender woman” or “transgender man,” because these specifications can imply that the person is not actually a man or a woman, which they are. These nuances can make it particularly challenging to determine how to frame queer-spectrum inclusive questions [21, 37]. “Conventional” gender survey questions (e.g. “please indicate your sex/gender: man, woman, other"), even when posited as capturing diversity, don’t capture the data necessary to know what identities are captured under “other,” and are, inherently, othering. Furthermore, most surveys don’t include questions about sexual, romantic, and related orientations, even though people with these identities experience identity-specific bias and discrimination and therefore should be explicitly considered [18].

Despite these challenges, as discussed earlier, population-level data from national surveys, including the National Family Growth Survey (NFGS), the US census, and the American Community Survey demonstrate both the increasing willingness of people to answer inclusive sexual orientation survey questions and the importance of how questions are framed [33, 34, 38, 39]. For example, the number of queer-spectrum respondents in the NFGS more than doubled in the 2015–17 survey, with either 7.6 or 8.6% of respondents identifying as gay, lesbian, or bisexual, depending on how the question was asked [39].

Even though younger people are more likely to identify with queer-spectrum identities [33, 34], so there are actually more people who identify within the queer-spectrum as time passes, it is likely that these types of rapid changes are largely caused by an increase in willingness to disclose queer-spectrum identities. These rapid changes in survey response numbers, along with the variation in response rate depending on how questions were asked, emphasize the need for regularly updated research-based survey questions to guide demographic collection practices.

#### Risks of self-identifying

While the United States Supreme Court case Bostock vs Clayton County, Georgia made gender identity and sexual orientation protected identities in the workplace nation-wide in the United States [40], people with queer-spectrum identities are still not broadly protected legally, and legal protections do not prevent all discrimination. Queer-spectrum people may also face severe repercussions if friends or family find out about their identity, such as being ostracized or disowned, and may also feel vulnerable due to historical practices of using demographic information to target people with marginalized identities. These risks were exemplified in a study by Villarroel [31], who found that people were more likely to select a queer-spectrum identity in a survey administered by a computer than by another person, and that this difference was greater in geographical areas with a less queer-inclusive political climate.

### Existing research on queer-inclusive surveys

It is important to distinguish between (a) sexual, romantic and related orientation identities and (b) gender identities because expression of these identities among college students impacts academic experiences (as well as life experiences more holistically), differently [41]. However, it was only recently that researchers began making a distinction between these two different types of identities and including those with queer sexual, romantic, and related orientations in social science research. Agreement on measurement and conceptualization of sex, gender, and sexual, romantic, and related orientation identities is still lacking [41, 42]. This is complicated by the multidimensional and fluid nature of these identities [42–44], and several researchers have proposed multidimensional scales for assessing sexual, romantic, and related orientations [e.g. 45]. But, these multidimensional scales are not structured for STEM education surveys that must be brief and whose demographic data is only a subset of a typically larger instrument.

Currently, if queer-spectrum identities are included in more general surveys (i.e. ones not focused on gender and/or sexual, romantic, and related identities), these identities are often lumped together (e.g. with LGBT, or lesbian, gay, bisexual, and transgender), erroneously implying that all people with different queer and intersectional identities have similar needs and experience similar challenges [35, 46, 47]. To the contrary, because individuals with different identities face different structural and social barriers within society, it is vital to collect information on both general (e.g. queer gender and/or queer sexual, romantic, and related orientation identity) and specific sex/gender (e.g. agender, genderqueer, transgender) identities [35, 41]. Furthermore, there is no indication of a general chronological progression towards change in how these data are collected, such as the papers discussed in this paragraph, with very recent papers still lumping identities together.

The existing suggestions and practices we found for inclusive survey questions ranged widely and included adding “other” to a binary question, providing an open-ended space for gender, including a multiple-choice list of gender options, or using a multi-question series [15, 18, 24, 28, 30, 36, 37, 39, 48]. Multiple-choice lists of gender options ranged widely, including options such as transgender, FTM/transmasculine; transgender, MTF/transfeminine; genderqueer, non-binary gender, agender/do not use label, questioning, and other [28]. Surveys specifically targeting transgender individuals used longer lists than general population surveys, and included terms such as: part time as one part time as another gender, gender variant, genderqueer, androgynous, feminine male, masculine female or butch, aggressive, third gender, drag performer, and two spirit [35]. While these identity-targeted surveys are important for certain types of research, and may be helpful in expanding existing overly-narrow general population surveys, demographic questions for a general population survey likely need a middle ground that creates an inclusive environment while still keeping the number of options provided manageable for respondents and for subsequent data analysis [49].

Multi-question survey formats, which were often rooted in medical fields [28, 50], tended to go beyond what is appropriate for a general or non-medical survey. For example, Tate et al. [30] recommend a two-question method; the first asked for current gender identity and the second, gender category assigned at birth (the study used gender not sex assigned at birth). While this format is helpful in some situations, particularly related to health records [50, 51], in many cases it is not relevant or appropriate to ask sex/gender assigned at birth, as this is often considered private information and undermines an individual’s gender identity when their sex assigned at birth is different from their gender.

A slightly different multi-question approach was to ask how an individual currently perceived themselves, and then, how others currently perceived the individual, on a scale ranging from feminine to masculine (e.g., ranging from 0, not at all, to 6, very), before asking for sex at birth (choose from a list) [23]. A major limitation of this format is that it places gender on a masculine-feminine spectrum, instead of allowing for a wider breadth of identities. Furthermore, by focusing on how others perceive the individual, it can undermine their gender identity by bringing attention to disparities between the individual’s self-perceptions and others’ perceptions, and it does not necessarily consider that an individual may be perceived differently in different social contexts. Another version of the multi-question survey uses yes/no questions and probes with additional questions in the case of “yes” answers [24]. While these types of multi-question formats can be useful for surveys specifically about gender, they add significant and often undesired length when one is simply attempting to collect demographic information. Similarly, questions about participants’ gender expression and how comfortable they feel expressing their gender may be important in some environments, as gender and gender expression are different constructs. While both gender and gender expression are performative, the latter is much more highly changeable and is also likely beyond the scope of the general demographics questions needed for most general STEM education research.

The range in inclusive survey types and underlying goals, such as separating cisgender and or heterosexual people from queer-spectrum individuals, asking about a range of queer-spectrum identities, or asking people to place their identity on a range of spectrums (e.g. male-female, asexual-sexual, and static-fluid [15, 24, 28, 35, 52, 53], demonstrates a lack of consistency in guidance for collecting queer-spectrum demographic information. Even though these existing surveys provide a range of options for collecting data, problems with these surveys that limited their applicability to general demographic data collection included a) a focus on collecting medical data, b) a lack of research basis or findings that conflicted with other peer reviewed literature, c) questions focused on a narrow set of genders or sexual, romantic and related orientations, or d) relying on identity spectrums that problematically assume that identity is linear.

One key piece that was missing from nearly all surveys targeted at a general population (i.e. not queer-specific), were questions that collected both gender identity and sexual, romantic, and related orientation data from the same people. Collecting disaggregated queer-spectrum identity data along with a range of other social identities can provide valuable information about people’s experiences, as identity is not additive—i.e. the experiences of someone with both a queer gender and a queer sexual, romantic, and related orientation cannot be extrapolated by adding the experiences of those with only a queer gender or a queer sexual, romantic, and related orientation [47]. To effectively address the experiences of queer-spectrum individuals, it is vital to create space for each individual’s multiple and interacting social identities, including both their gender as well as their sexual, romantic, and related orientation identities.

### Research questions

To address the two-pronged problem of both a lack of broadly applicable queer-inclusive demographic questions and a lack of information on the under-representation of queer-spectrum students in STEM, we: a) developed and implemented queer-spectrum inclusive demographic questions in our existing study of engineering and computer science undergraduate students, and b) compared the results from these questions to national data sets and data we had previously collected in the same courses using a more conventional gender identity question to address the following research questions:

1. Are students with a) queer genders and b) queer sexual, romantic or related orientations under-represented in the undergraduate computer science and engineering courses, relative to national data?
2. Which of three ways of asking gender demographics questions, conventional (man, woman, other), queer-inclusive (a range of identity options including a space to self-identify), and fully open-ended (no choices, just open response), produce the most informative data regarding students’ queer identities?

## Materials and methods

### Researcher contexts

This study was performed by a team of STEM education researchers, including an associate professor in civil and environmental engineering, a professor in biology, and a STEM education research scientist who has a background in biology, ecology, and engineering education. The civil and environmental engineering associate professor and research scientist are both involved in the larger research project of which this study is situated. Within our research team queer gender; queer sexual, romantic and related orientations; cisgender; and heterosexual identities are represented.

### Course contexts

These data were collected as part of a larger study aimed at developing, implementing, and evaluating course activities to better prepare engineering and computer science students to recognize the contributions of diversity to their professional fields, to work inclusively with others, and to consider the societal implications of their technical work [54]. All research was approved by the Institutional Review Boards at each institution, and we obtained written consent from all participants.

All of our data were collected in computer science and engineering courses and most of the data were collected from surveys of first-year students in various introductory courses. At the Mid-Atlantic Public university, data were collected from general first year classes taken by all students with engineering majors (n = 2957) as well as upper-level engineering courses (n = 294), with one student who was in both an upper and lower course. At the Rocky Mountain Public university data were collected from major-specific introductory engineering classes (Mechanical n = 436, Civil and Environmental n = 351, Chemical and Biological n = 300, Electrical and Computer n = 369, an engineering open-option course n = 194, and course unknown n = 95), and senior design students (n = 443). At the Rocky Mountain Private University, data were collected in both lower-level (n = 132) and upper-level (n = 163) engineering classes, as well as lower-level (n = 53) and upper-level (n = 34) computer science classes. Additionally, 162 computer science and 39 engineering students did not specify the level of their course. At the Rocky Mountain Public Teaching University data were collected in first-year computer science courses (n = 44), and upper-level computer science courses (n = 30). Students who were enrolled in multiple participating courses within the same semester and who consented to participate in the study only completed the survey once. Data were also checked to remove any duplications for students who completed the survey in different courses in different semesters, such that each student is only represented in the data once, even if they completed the survey multiple times.

Because the larger research project is focused on diversity in engineering and computing and different students’ experiences, including gender-related experiences, we were interested in determining if we could better capture participants’ gender identities, beyond the initial “male, female, other” scale that researchers used at the beginning of the study. The full surveys our demographic questions were a part of included a variety of different scales intended to measure the impact of the new course activities on constructs such as students’ self-efficacy, belonging, appreciation for diversity, and intent to enact inclusive behaviors [54].

While there is not a consensus in the research on the best practices for locating demographic questions in survey (see Huges et al., [18] for a synthesis of recent research), Gilovich et al [55] recommends placing demographic questions at the beginning of a survey if demographic information is important to the survey and Huges et al. [18] further point out that placement of demographics at the beginning allows researchers to identify demographic patterns for those who do not complete surveys. Therefore, the demographic questions discussed in this paper were usually included at the beginning of the first survey of the semester to meet the needs of the larger project.

The overall percent of students who both completed the survey and consented to have their data included in our study was 70–80% within a given course. In some courses students received homework or extra credit for the survey and students had the option to complete the survey but not consent to have their data included in the study. Because students were potentially enrolled in multiple courses both within the same semester and across semesters, and we do not have headcounts of those who either did not complete the survey or completed the survey but did not consent to participate in our study, we cannot calculate an exact survey response rate. Even without an exact response rate, a 70–80% response rate is well above the ~50% mean and median survey response rates found in a meta-analysis of survey study response rates, including the mean response rate of 49% for survey studies performed in the field of education [56].

### Survey development and implementation

In our study we report on three different survey types that collected gender identity information: a conventional survey, a queered survey, and an open-ended survey, which were administered during different semesters across the different institutions. The conventional survey was not designed to be part of a research project on different ways to write survey questions that collect gender information, it was simply how the researchers developing the survey for the project wrote the survey question to collect gender information from participants. As such, it is representative of how researchers often conventionally write demographic questions. These conventional survey questions were administered as part of the survey for the larger research project during the 2017–2018 academic years. In 2017 this survey included a conventional gender identity request “please indicate your sex,” and provided the options “male” or “female.” For the Spring 2018 survey the options were revised to “male,” “female,” “other,” and “prefer not to respond.” These two slightly different survey versions are referred to as “conventional,” as they represent ways that these demographic questions are commonly asked.

As part of an internal review of the project, prior to the administration of the Spring 2019 surveys we revised the question about sex/gender to a multiple-choice list, which also included a “prefer to self-identify” open-response option (Box 1).

**Box 1. Gender and sexual, romantic, and related orientation survey questions**

Please indicate the gender(s) you affiliate with.

        Female/Feminine
        Genderqueer/Genderfluid
        Intersex
        Male/Masculine
        Nonbinary/Third gender
        Transgender
        Two-spirit
        Prefer not to respond
        I don’t understand the question
        A gender not listed here ___________________

Please indicate the sexual orientation(s) you feel describe you most closely.

        Asexual
        Bisexual
        Gay
        Lesbian
        Pansexual/Omnisexual
        Straight or heterosexual
        Queer
        Prefer not to respond
        I don’t understand the question
        A sexual orientation not listed here __________________

This major revision of the demographic questions was based on the literature that discusses other existing surveys [e.g. 24, 57–60], as well as iterative discussions and feedback with queer-spectrum, cisgender and heterosexual colleagues and content experts who also had a range of racial/ethnic identities and who lived in a range of geographical areas throughout the United States. Thus, through iterative revising and consulting with content experts with a range of identities we developed and piloted our survey prompt and responses. We used a similar process to develop a separate survey question about sexual, romantic, and related orientations, which had not been previously asked. At the end of the full set of demographic questions we included an open-response question in which students could provide information about any identity not already asked about.

These iterative discussions exemplified the challenges in developing a survey with queer-inclusive language, particularly since colleagues in different regions of the United States had different opinions of appropriate language, including around the seemingly simple categories of “man” and “woman.” Our use of “Female/Feminine” and “Male/Masculine” is representative of concerns raised by others who had worked with transgender students in survey development and reported that transgender students did not necessarily think “woman” or “man” inherently included them.

Similarly, the language of the prompts, including “the gender(s) you affiliate with” was specifically crafted in response to concerns of those piloting the questions that using the language of “identify with” would be more likely to limit queer respondents’ choices to more narrow definitions of an identity category–narrow definitions they themselves may not use, but that they perceive are used by others. As such, we followed the practice of centering those with marginalized identities in our survey construction, rather than centering majority identities and conventions that may be exclusive in developing our prompt text [61, 62]. This survey is referred to as “queered”.

Once we developed our revised survey, we carefully considered how to order the choices for both the gender and sexual, romantic, and related identities questions, as option order can influence how participants respond [49]. We unfortunately were unable to find explicit guidance about ordering these types of questions. However, we did not want to follow the convention of listing the binary options first (e.g. [24]), as through listing commonly found answers first respondents may not fully read the answers and realize that there were additional options they were not used to seeing on surveys, but that fit their identities better [49]. Due to a lack of more detailed guidance, we listed the options in alphabetical order for both questions, with the non-response options (e.g. prefer not to respond or do not understand the question) listed last. While future research on question ordering would be helpful, our order strategy at least allowed us to avoid biasing the responses towards the commonly found options on a gender survey [49].

After we administered the queered questions during the Spring 2019 semester, the gender question was changed to an open-ended format for the Mid-Atlantic Public university, using the same prompt as the queered survey–“please indicate the gender(s) you affiliate with” and the sexual, romantic, and related orientation question was removed. Students were still provided with the open-response question that asked about any identities they wanted to provide which had not been specifically asked about.

This change was made at the Mid-Atlantic Public university because those who were working with the Mid-Atlantic Public university were concerned that the more explicitly queer inclusive question would negatively impact the perceptions of other collaborators at the university, as well as of the participating students about the project as a whole. These concerns were based on the few sarcastic or hostile responses that students provided on the queered survey, even though these types of responses were also provided by students at other universities on both the new queered survey as well as by students on the previously used conventional survey. Thus, this change was primarily in response to concerns about how these inclusive questions might harm relationships with those at this university and student perceptions of the project, rather than because there was a large negative response to the questions when they were implemented. To keep the survey questions as consistent as possible, the researchers involved in changing the questions simply removed the options and did not revise the prompt text. Therefore, while this survey variation was not designed to test different ways of asking questions about gender, it does represent the complex social contexts that researchers perform research in. This version of the survey is henceforth described as “open-ended”. The type of survey administered by semester and institution is described in Table 1.

**Table 1:** Survey type administered by institution and semester.

Institution	Student Level	Conventional n = 2542	Queered n = 1932	Open-ended n = 1633
**Mid-Atlantic Public**	First Year	S18, F18	S19	F19, S20
**Mid-Atlantic Public**	Upper Level			F19, S20
**Rocky Mtn. Private**	First Year	F17, F18	S19, F19, S20	
**Rocky Mtn. Public**	First Year	F17, S18, F18	S19, F19, S20	
**Rocky Mtn. Public**	Senior		F18, S20	
**Rocky Mtn. Public Teaching**	Introductory		F19, S20	

While the survey questions evolved over time as the larger research project matured and we did not initially intend to compare how students responded to different demographic questions, we realized that our dataset provided a unique opportunity to learn more about how students responded to different demographic question types and that this information could be useful to other STEM education researchers. Because these questions were part of a larger survey that was designed to be used to compare student responses to other questions, this post-hoc analysis of our data is still a robust way to analyze our data. As an additional check, we compared institutionally collected demographic data about students in each department or college of each institution where we collected data and found that the student populations were very similar across the three years that we collected data (see S1 Table for details). These similarities across years indicate that the groups across time were similar enough to combine in a single analysis.

To further systematically address concerns about secondary analysis of data we considered how our data addresses Engel and Schutt’s six questions that one should ask before using secondary data, as discussed in DeCarlo [63]. In summary, our data were collected with the intent of collecting the same information that we used the data for, in a continuous and relevant timeframe, and using Qualtrics surveys by Ph.D. level researchers with extensive education research experience. Additionally, as two of the authors on this paper are involved in the larger project, we had direct access to the data output by Qualtrics without curation of missing data and we had no barriers to obtaining information about the data or data collection process. See S2 Table for a more detailed description of both Engle and Shutt’s questions and how our data address these concerns.

### Statistical analyses

Statistical analyses were performed in R version 3.6.2. All statistical comparisons were made using a two-sided Fisher’s Exact Test [64]. In our comparisons, described in the results section, we compared the number of students who selected a queer gender identity or a queer sexual, romantic, or related orientation with the number of students who would have had those identities, had representation been proportional based on existing population level statistics for queer gender identities and orientations. As such, in both of these cases we had a 2x2 table of expected and actual results, which is consistent with the use of a Fisher’s Exact Test.

To determine the expected number for the population-level comparisons we used the best and most recent available data that provides similar queer gender and queer sexual, romantic, and related orientation data disaggregated by age; the data collected by Harris Poll and Gallup [33, 34] that we discuss in our introduction. Because of the lack of widely established and accepted population numbers for queer-spectrum individuals, and the Harris and Gallup surveys collected data using different categories, we compared the specific category results that overlap between the two surveys (bisexual, gay or lesbian, and transgender) to evaluate the consistency between the two polls when determining how to calculate the expected population-level numbers for our analyses (Table 2). Based on these overlapping categories, both polls had very similar percentages of transgender respondents, and the Gallup poll had nearly double the number of bisexual and gay or lesbian respondents. Even though the Harris Poll found that overall 20% of the 18–35 years identified as LGBTQ and the Gallup Poll found that 16% of generation Z (born in 1997–2002) identify as LGBT, these differences in population percentages appear to be primarily due to differences in how questions in each survey were framed, and demonstrate that providing more identity categories may lead to more people identifying on the queer spectrum. The Gallup Poll also had an “other” category to capture queer identities beyond LGBT. However, this category only had a 0.4% response rate, in contrast to the much higher response rates of the additional categories included in the Harris Poll—sexual orientation: 4% asexual, 2% pansexual, 1% questioning, 0% queer; gender: 3% agender, 3% gender fluid, 2% transgender, 2% unsure/questioning; 1% bigender, 1% genderqueer [33, 34]. Therefore, because the Harris Poll survey questions were more similar to our survey questions and because our comparison shows that these estimates are similar or more conservative than the Gallop poll numbers, we chose to use the Harris Poll data to calculate our expected numbers for both groups of queer individuals for our analyses.

**Table 2:** A comparison of population-level data on those with queer-spectrum identities in the 2017 Harris Poll (n = 2,037) and 2020 Gallup Poll (n>15,000 across all age groups) [33, 34].

Identity Category	Harris	Gallup Generation Z
	Age 18–35	
**Bisexual**	6%	11.5%
**Gay or Lesbian**	2%	3.5%
**Transgender**	2%	1.8%

This table provides a comparison of the results of the Harris and Gallop polls for the identity categories for which both surveys collected data and does not include the categories that did not overlap between the two polls.

We also made comparisons between the proportion of students who selected a queer gender identity on the conventional survey and those who selected a queer gender identity on the queered survey. These comparisons were also 2x2 table comparisons, comparing the two different survey types. Lastly, for our comparison of the under-representation of those with queer identities to women in engineering, we compared the actual number versus expected number for individuals with these two different gender identities. As is convention for reporting Fisher’s Exact Test statistics, only p-values are reported, as there is neither a specific test statistic to report nor degrees of freedom.

## Results

Overall, we found that students with queer gender identities and queer sexual, romantic, and related orientations were under-represented in our undergraduate engineering and computer science student dataset compared to similar age cohort general population data [22, 33]. Students were significantly more likely to report a queer gender identity in the queered survey than in the conventional survey that included an ‘other’ option. In addition, students were significantly less likely to leave the queered gender survey question blank than the fully open-ended gender question. Overall, we found that all of the queer gender and sexual, romantic, and related orientation identities students provided in any of the open-response boxes (i.e. the self-identify options in the conventional and queered survey, as well as the open-ended survey) were either already included in the queered demographics options, or could easily be included with minor revisions to the questions. Therefore, the queered demographics questions provided the most robust way to capture students’ identities, both in terms of response rate and students revealing their queer identities in their responses.

### Under-representation

#### Gender

Based on our queered and open-ended surveys, students with queer gender identities made up 0.84% of our respondents. Therefore, they were significantly under-represented when compared to the 12% of the US population aged 18–34 (the age-range of nearly all of our students), who identify as a gender other than cisgender [33] (Table 3; p<0.0001). This under-representation is so stark that students with queer gender identities in our study are only 7% of the expected number, if representation were proportional. While students within each institution may represent different engineering and computer science related majors, as well as other differences, there was a large range in the percent of students with queer gender identities by institution, from 0.45% to 2.58% (Table 3). Even at the institution with the highest percentage of queer gender identities, these students are still less than ¼ of the expected number of students, if representation was proportional. Considering the range in sample size and the small percent of individuals with queer gender identities, some of this variation, particularly at the RM Public Teaching institution, where n = 74, may be due to relatively small sample sizes, yet these small sample sizes still do not explain the drastic under-representation shown in our data. Due to this variation in sample size and variation in the types of surveys administered we do not make statistical comparisons across institutions.

**Table 3:** Queer-spectrum and cisgender/heterosexual student identities.

	Gender			Orientation		
	N	Queer (%)	Cis (%)	N	Queer (%)	Heterosexual (%)
**Overall**	**3698**	**0.84**	**95.16**	**2131**	**11.49**	**81.66**
Mid-Atlantic Public*	2236	0.45	94.14	603	8.94	86.09
RM Private^†^	310	2.58	97.10	310	11.61	84.52
RM Public^†^	1078	1.05	96.47	1078	12.76	78.13
RM Public Teaching^†^	74	1.35	97.30	74	12.16	81.08

Percentages may not add up to 100% due to responses such as "prefer not to respond," "I do not understand the question”, or blank answers.

*: S19 data are from queered survey, F19 and S20 data are from open-ended survey

†: Data are all from queered survey

In addition to being under-represented overall, in our study those with queer genders were proportionally more under-represented than women. In the queered survey, 27.7% of the respondents were cisgender women (Table 4; 0.7% of those who selected Female/feminine were not cisgender, as they selected multiple gender identities). This percentage of respondents is slightly higher than the population of women shown in the institutional data in S1 Table, not unexpected as women are more likely to complete surveys [65]. Given that women make up 50.8% of the US population, women are under-represented by a little less than ½ in our data set (or a little more than ½ based on institutional data); in contrast, those with queer identities were 7/100 of that expected based on national data (p<0.001). We note the percent of women in engineering is known to vary by discipline, and therefore the percent of women in engineering will potentially vary with the courses that are surveyed [66]. There are not enough available data to say if representation of queer-spectrum individuals also varies across engineering discipline.

**Table 4:** Queered gender across all institutions, based on the queered survey (n = 1932).

Please indicate the gender(s) you affiliate with:	%
Female/feminine	27.95
Genderqueer/genderfluid	0.41
Intersex	0.05
Male/masculine	69.57
Nonbinary/Third gender	0.41
Transgender	0.36
Two-spirit	0.16
Prefer not to respond	0.78
I don’t understand the question	0.83
A gender not listed here:*	0.05
Left blank	0.15

*: The one gender response given was “*neutral*, *but biologically female*,” which is reported as 0.05%. This option also garnered responses that were not genders (e.g. *“there are only two genders*.*”*) Therefore, if both gender and non-gender responses are included, this is 0.88%.

#### Sexual, romantic, and related orientations

Students with queer sexual, romantic, and related orientations made up 11.04% of our respondents, and were also under-represented, when compared to the 16% of the US population aged 18–34 that is non-heterosexual [22] (p = 0.0003; Tables 3 and 5). These data published by GLAAD were collected in 2016, so the GLAAD cohort is now 22–38 [33]. Therefore, if anything, our students are slightly younger than the GLAAD cohort, and overall both the GLAAD survey and the Gallup Poll survey found that younger people were more likely to report queer identities [33, 34]. Similarly, the 7% students in our study with bisexual, gay, and lesbian identities are significantly underrepresented when compared to the 15% of people in generation Z with these identities in the Gallup Poll (p<0.0001) [34].

**Table 5:** Sexual, romantic, and related orientations across all institutions, based on the queered survey (n = 1932).

Please indicate the sexual orientation(s) you feel describe you most closely.	%
Asexual	3.99
Bisexual	4.71
Gay	1.40
Lesbian	0.78
Pan/Omnisexual	0.83
Straight/heterosexual	82.35
Queer	0.52
Prefer not to respond	2.69
Don’t understand	1.19
Self-identify	0.15*
Left Blank	2.12

*: The three responses that were not sarcastic or aggressive were demisexual (2) and biromantic and are represented by the 0.15%. When including sarcastic or aggressive responses to this category, it is 0.41%.

### Comparison of survey types

Our comparison of survey types focuses only on the gender surveys, as we did not have multiple survey types that collected data on sexual, romantic, and related identities. For the conventional and queered surveys, which were administered at all campuses, the queered gender survey (Table 4) provided a significantly higher response rate of queer gender identities than the conventional survey (Table 6). While the conventional survey including a fill-in “other” option did provide space for students to identify queer gender identities, this survey resulted in only 0.15% of students providing a gender identity beyond male or female. In comparison, the queered survey resulted in 1.13% of students identifying themselves as having a queer gender, significantly more than the conventional survey (p<0.0001). The conventional survey also limited students to selecting one option, and confounded sex and gender. In the queered survey, students were allowed to select multiple gender identities, and 78% of students who indicated they had a queer gender identity selected more than one gender identity (e.g. female/feminine and non-binary). The queered and conventional surveys had nearly identical non-response rates, of 0.15% and 0.20%, so adding queer options to the gender identity question did not decrease the percent of students who responded to the question.

**Table 6:** Conventional survey across all institutions (n = 2542).

Please indicate your sex	%
Male	72.54
Female	25.73
Other OR I do not identify as either. I identify as:*	0.15
Prefer not to respond	0.43
Left Blank	0.20

*: Only 4 (or 0.15%) of the “other” responses were genders: genderfluid, transfeminine, and non-binary. However, this option also garnered responses that were not gendered, such as “Attack Helicopter” and other sarcastic/hostile responses. Therefore, if both gender and non-gender responses are included it is 0.43%.

For the Mid-Atlantic Public university, the only institution where students completed the open-ended survey, there was no significant difference between the percent of students who identified as having a queered gender and those who did not for the queered and open-ended surveys (p = 0.3051). However, the open-ended question had a non-response rate of 5.57%, which is significantly higher than the non-response rate of data collected at the Mid-Atlantic Public university using the conventional survey (0.5%) or queered survey (0.2%; p<0.0001 for both comparisons; Table 7). This low response rate was not simply due to larger non-response patterns in the survey because all of these students: a) provided an answer to the immediately preceding closed-response race/ethnicity question, and b) answered survey questions that followed the gender question in ways consistent with those who did answer the gender question. These patterns indicate that students who left the gender question blank were not avoiding all demographic questions and did not simply stop responding to the survey just before the gender question. Therefore, this increase in the percent of blank responses at this institution when the prompt switched from queered to open-ended decreased the number of students whose data can be used when aggregating data by gender identity and indicates a decrease in response rate to this specific question.

**Table 7:** Consolidated responses from open-ended question at Mid-Atlantic Public university (n = 1633).

Please indicate the gender(s) you affiliate with:	%
Female/woman	19.53
Nonbinary/genderfluid	0.24
Male/man	73.55
Transgender	0.12
Questioning	0.12
Inclusive answer (e.g. everyone)	0.43
Sarcastic/aggressive answer	0.55
Heterosexual	0.49
Prefer not to answer	0.98
Left Blank	5.57

Because of the nature of the open-ended question, the open-ended question also had other types of answers that did not provide meaningful gender information, including 0.49% of students who provided a sexual, romantic, or related orientation (in this case all heterosexual), and 0.43% of students who wrote an inclusive answer, trying to demonstrate they were not sexist, *“I affiliate with all genders because I’m not transphobic or sexist”*. However, the response rates for these types of responses are similar to the “I don’t understand the question” response rate in the queered survey of 1.33% for the Mid-Atlantic Public institution, indicating no notable change in the percent of students who either self-indicated they didn’t understand or who provided a response indicating they didn’t understand the question.

At all institutions all survey types administered received a few answers that were sarcastic and/or aggressive, such as *“Apache Attack Helicopter*,*” “Popeye’s spicy chicken*,*”* and mild vulgarities, but these were <1% for all survey types. Like Haverkamp [67] we believe it is important to report malicious responses because they reflect the lived experience of those with queer-spectrum identities. Haverkamp [67] specifically argues that these responses reflect the social and education contexts in which our students are embedded.

### Representation of queer identities

The queered surveys captured nearly all of the gender and sexual, romantic and related orientation identities provided in either the “self-identify” option in the queered surveys, or in the open-ended survey (Tables 4, 5, and 7). In the open-ended gender question, a few students included a straight/heterosexual sexual orientation, conflating and/or combining gender and sexual, romantic, and related orientation in their responses.

The surveys with the open-ended gender question did not have a sexual, romantic, or related orientation question. However, some students provided their sexual, romantic, or related orientation in the question asking about any other identities that influence their experience as students. Unlike those who included a sexual, romantic, or related orientation in the gender question, those that included a sexual, romantic, or related orientation in the “any other identity that influences your experiences” question were almost exclusively queer. As a metric to provide initial evaluation of the sexual, romantic, or related orientation options, the sexual, romantic, or related orientations provided in the “any other identity that influences your experiences” question were also well represented in the sexual, romantic, or related orientation question in the queered survey.

In evaluating responses to the queered demographic questions, there were no identity options with a 0% response rate for either survey (Tables 4 and 5). Additionally, students made use of the ability to select multiple options within a single survey question, with 78% of those with queer gender identities selecting two or more options (e.g. female/feminine and non-binary), and 9% of those with queer sexual, romantic and related orientations selecting two or more options (e.g. asexual and lesbian).

## Discussion

Inequities for and discrimination against queer-spectrum individuals abound throughout the US, yet there is a growing body of literature demonstrating that queer students and professionals face greater inequities in STEM than in other fields [1, 4, 7, 12, 15]. Inclusion of both queer gender and queer sexual, romantic, or related orientation identities in demographic questions is a first vital step towards addressing these inequities–both within and beyond STEM—yet research that includes queer-spectrum individuals continues to be lacking, in part because queer-inclusive questions need to be used whenever demographic questions are asked, not simply when researchers have a specific interest in queer identities. Through collecting queer-inclusive data, our data are some of the first to demonstrate the under-representation of both queer gender and queer sexual, romantic, or related orientation students in undergraduate engineering and computer science classes across multiple institutions. Data such as ours need to be collected in other STEM fields to provide more information about representation of queer-spectrum individuals in other STEM fields. One cannot simply assume under-representation of queer-spectrum individuals will parallel that of women in STEM.

Existing research is mixed on how well the gender parity for men and women in a STEM field predicts the presence of queer-spectrum individuals, and thus the patterns of under-representation in our data may be a systemic STEM-wide problem and may follow different patterns than the patterns of under-representation of women [1, 5, 20]. Yoder and Mathesis [26] found that the percentage of women in a field was correlated with the likelihood that lesbian, gay, or bisexual individuals disclosed their queer identity to colleagues, which is different from predicting overall numbers of LGB individuals, but does provide some information about climate. Sansone and Carpenter [32] found a higher number of men in same-sex couples in STEM fields that have higher proportions of women. But, conversely, Malory and Hughes [5] found the lowest representation of trans and non-binary students in biology classes, and Cech And Waidzunas (2021) found no relationship between representation of queer-spectrum individuals and STEM field. Therefore, it would be erroneous to assume that underrepresentation is a problem limited to engineering and computer science or that the under-representation of queer-spectrum individuals inherently parallels the underrepresentation of women in STEM.

Through demonstrating the presence and under-representation of queer-spectrum engineering and computer science students in our study, we help demonstrate the need for queer-inclusive practices and programs that will support current queer-spectrum students, create a more supportive and equitable environment for future queer-spectrum students, and create cultural change such that all people value creating a supportive and equitable environment. Our data also support the growing list of literature that both calls for queer-spectrum identity inclusion both within and beyond STEM and demonstrates that students respond to queer-spectrum identity questions or include their queer-spectrum identities when given the space to do so [4, 15, 17, 21, 22]. These types of responses are congruent with Broussard et al. [21], who found that those with queer-spectrum identities overwhelmingly wanted demographic questions with queer-inclusive options, and also help reinforce alternative queer-inclusive narratives, countering exclusive dominant, or master, narratives [17]. Furthermore, inclusion of queer-spectrum identities in routine demographic questions normalizes queer-spectrum identities and counters existing dominant narratives, rather than enforcing these narratives and literally othering those with these identities, either through an “other” box, or ignoring them altogether [17, 18].

Our findings contribute to the development of queer-inclusive surveys by clearly framing the existing problem and providing initial research-based gender and sexual, romantic, and related orientation questions, which can serve as the basis for future studies within and beyond STEM. Because surveys that are not inherently queer-spectrum inclusive are one of the ways narratives that exclude queer-spectrum people are enforced, a large-scale shift towards inclusive demographics questions also works to create more inclusive cultural narratives regarding societal gender and sexual, romantic, and related orientation requirements and norms [19].

### Social and cultural contexts

When we work to create queer-inclusive spaces through a range of approaches, including queer-inclusive demographic questions, it is important to consider the larger social and cultural context of the respondents. In our data, the Mid-Atlantic Public institution had notably fewer students who self-reported both queer gender identities and queer sexual, romantic, and related orientations than the schools in the Rocky Mountain region. However, the two different geographies represented in our study have notably different political orientations, as exemplified by the differences in legal protections for those with queer-spectrum identities in these locations [68]. The Rocky Mountain region institutions are in a state ranked much higher for its legal protections for those with queer-spectrum identities than the Mid-Atlantic institution [68]. While it is possible that there are simply fewer people with queer-spectrum identities at the Mid-Atlantic institution in engineering and computer science majors, with queer-spectrum people seeking out institutions in locations with relevant legal protections, the south-eastern United States contains the highest percentage of the queer-spectrum population, despite collectively having the most hostile political climate for queer-spectrum people [69].

Therefore, our results lead to the question: what is it about the environment––of not just the institution, but also of the broader community in which the institution is embedded––that leads to this under-representation? Because our data collection methods required queer-spectrum people to reveal their identity, it is also possible that the difference in our results is due to differences in self-reporting, rather than population differences. In this situation, additional research could potentially reveal if people are under-reporting queer-spectrum identities.

Students, particularly those with multiple marginalized identities (e.g. queer-spectrum students of color and/or those with a disability) navigate a challenging juggling act in college as they navigate if and how to disclose their concealable identities, as they are also navigating their own identity development [70, 71]. Willingness to disclose identities on the surveys may also be interacting with their identity development. However, since national data shows that, as a whole, younger individuals are more likely to report queer-spectrum identities on surveys [33, 34], one cannot simply explain the under-representation we found in our data with college -age students’ identity development causing an unwillingness to disclose their identity. While willingness to disclose queer-spectrum identities may be influenced by students’ major, the low reporting we found would still point to problematic exclusive environments for engineering and computer science majors.

To address challenges around identity disclosure, participants might feel more comfortable reporting identities in a fully anonymous survey; this would be consistent with Villarroli [31], who found that there was a bigger increase in the proportion of people reporting queer-spectrum identities on their computer-administered survey than in their phone-administrated survey in more conservative regions of the United States. However, this type of survey could only confirm under-reporting and could not confirm any other conclusions. A survey disseminated through a place like a campus queer center might lead to a higher response rate but would not give us data comparable to our original survey [72].

It is also possible that students with queer-spectrum identities in engineering and computer science majors are both under-represented and under-reporting at the Mid-Atlantic institution, due to lower queer-spectrum student enrollment and a lower likelihood to self-report queer-spectrum identities, which could be caused by the less supportive climate of the region where the university is located. This is also the institution where the queer-inclusive questions were administered for only one semester, after which the open-ended survey was used. This change, along with not asking the sexual, romantic, and related orientation questions, came out of concerns about how the project would be perceived by respondents and others interacting with the project.

This removal of queer-inclusive questions is an example of how decisions that are driven by fear of backlash from a dominant perspective can have unintended erasure effects. For example, when excluding questions about sexual, romantic, and related orientations is considered a normal and therefore neutral practice, the harm done by this normative erasure of queer-spectrum people is likely not considered by cisgender and heterosexual individuals.

### Survey types

The inclusion of queer-spectrum identities in routine demographic questions normalizes queer-spectrum identities, creating a more inclusive environment and helping to counter exclusive cultural norms. However, a major challenge in creating queer-spectrum inclusive demographic questions is determining how to frame these questions. There are a range of options suggested in the literature, however there is still not a large-scale research-informed set of demographic questions, unlike those that are available for race and ethnicity [73]. Two simple and common strategies currently used are to either add an open response option (e.g. “other”) to a conventional male/man and female/woman question, or to simply provide an open response box for respondents to fill in.

Our results demonstrate that both of these options have limited effectiveness capturing useful data on queer identities: significantly more students provided queer identities in the queered survey than the conventional survey, with both survey types having near 100% response rates; and, while there was no difference in the percent of students who identified as having queer gender identities for the queered and open-ended surveys significantly more students (>5%) simply left the gender question blank in the open-ended survey. This higher non-response rate in an open-ended question is commonly found in survey research, as these types of questions require respondents to generate their own material instead of simply checking a box, leading to a higher cognitive burden for respondents [49]. Because the open-ended question was only administered at the Mid-Atlantic Public university, it is possible that this decrease in response rates would differ at other institutions. However, since this decrease in responses is consistent with existing research about open ended survey questions in general, it is reasonably likely that this pattern would be found in at least some other institutional settings as well. Therefore, we recommend using identity questions with multiple, queer-inclusive options, which include a fill-in ‘prefer to self-identify’ response and allow respondents to select multiple options.

Beyond a lower response rate, the conventional survey with a write in “other” option had additional problems around inclusiveness, as it still largely centers binary identities and lumps all queer gender identities together, aligning with a master narrative that norms cisgender and heterosexual identities [17]. Conversely, a list that includes a range of gender identities and allows respondents to select multiple options shifts away from privileging only binary identities and allows respondents to communicate complex identities, countering the dominant narrative of cisheteronormativity [19]. As part of this alternative narrative, the inclusion of these queer identities as check-box options also makes a clear inclusivity statement supporting queer-inclusive narratives, which is also often explicitly in alignment with current diversity and inclusion initiatives in STEM.

While it may seem simpler to include only a few queer identities along with other option in a demographic survey, a practice recommended by the GenIUSS Group [24], data from the recent Harris and Gallop Polls demonstrates that this practice still provides limited information on queer identities. As we discussed in the introduction, the differences response rates between the “other” category used in the recent Gallop Poll and the range of additional queer identities in the Harris Poll indicate that providing a few queer identities and an “other” option has a limited ability to capture queer identities not listed in the survey [33, 34]

A fully open-ended survey question may seem like the best option, as it easily provides space for respondents to write whatever they want. However, a fully open identity question also has limitations beyond the aforementioned increased cognitive burden on survey takers and lower response rates [49]. When used to ask questions of identity, a major shortfall of a fully open-ended question is that it requires researchers to re-classify identity responses before they can be used in quantitative data analysis. This practice has ethical problems, as it shifts the power of identification from the survey respondent to the researcher. Instead, by providing respondents with categories, each individual has the agency to interpret their own identity through the options provided. If the survey data will eventually be used to bin responses into specific identities, each respondent should have the agency to pick their own identity “box,” rather than being classified by a researcher. This may be one way that critical researchers can attend to power dynamics inherent in the relationship between researcher and researched to better expose potential bias imposed by investigators, a strategy long recommended by feminist researchers [74].

Providing space for participants to select their own identity “boxes” is also practical, in that attempting to re-classify respondent identities into researcher-defined boxes is at best difficult even for someone well-versed in different queer-spectrum identities. While some re-categorization by researchers may be necessary after data collection, this re-categorization can be explicitly defined by the identities participants chose. For example, in Table 3 of this paper, we grouped all non-cisgender students (i.e. all students who picked a gender other than a single binary gender option) and all non-heterosexual students (i.e. all students who picked a sexual, romantic, or related orientation other than only “straight/heterosexual”). But, through our data collection methods we are able to provide this coarser-grained information and simultaneously provide a finer grained description of the students who selected these identities and avoid creating the impression that all people with queer gender identities and/or sexual, romantic, and related orientations have monolithic experiences [35].

In addition, similar to the conventional survey, unless queer-spectrum identities are explicitly named or included in the framing of an open-ended survey question, students may perceive that those who designed the survey are not actually inclusive of queer-spectrum identities, effectively supporting existing non-inclusive master narratives around queer-spectrum identities [75]. Additionally, the identities students provided in the open-ended question were almost all represented in the queered demographic survey, and those that were not well represented can be easily added through minor revisions of the queered survey questions (see Box 2 for our revised survey and the text in S1 Appendix for these revised questions with recommendations for implementation).

**Box 2. Revised queer identities survey questions**

Please indicate the identity(ies) you feel most closely describe your current gender(s). Select all that apply.

        Agender
        Female, Feminine, or Woman
        Genderfluid
        Genderqueer or Non-binary
        Gender non-conforming
        Intersex
        Male, Masculine, or Man
        Not cisgender, but I don’t identify with a specific identity
        Questioning or figuring it out
        Transgender
        Two-spirit or other Traditional or Indigenous genders
        Prefer not to respond
        I don’t understand the question
        Prefer to self-identify ___________________

Please indicate the identity(ies) you feel most closely describe your current sexual, romantic, and related orientation(s). Select all that apply.

        Asexual or Ace spectrum
        Bisexual
        Gay
        Lesbian
        Not heterosexual, but don’t identify with a specific identity
        Pansexual or Omnisexual
        Questioning or figuring it out
        Straight or heterosexual
        Queer
        Prefer not to respond
        I don’t understand the question
        Prefer to self-identify __________________

Specifically, we added “agender” to the gender identities and added “questioning and/or figuring it out” to both questions, as well as an option to identify as not cisgender or not heterosexual without selecting a specific identity. We also made other minor revisions to the survey to help clarify or improve the response options. For sexual, romantic or related orientation, we had respondents write in “demisexual” and “biromantic.” However, as these are asexual-spectrum identities [76], we did not add additional categories based on these responses. We did revise the “asexual” category to “asexual or ace spectrum,” but also think of these responses as an example of respondents using the “prefer to self-identify” option as we intended when we designed the survey.

In our open-ended survey, we also had problems with lower response rates. While a lower response rate is common for open-ended questions [49], part of the high non-response rate may have been the framing of the question. Clearer open-ended text may increase the response rate with gender identity information [49]. However, changing the wording would not address the higher cognitive load required to respond to open-ended surveys [49] and without careful wording this clearer text would still likely fail to explicitly recognize those with queer-spectrum identities.

While it is not practical, nor arguably useful, to include all possible queer-spectrum identities as options, including a range of queer-spectrum identities on the survey potentially communicates a shift in the power relationship between researcher and subject even for students who choose the “prefer to self-identify (blank)” option, through providing queer-spectrum individuals with the agency to determine how their identity is represented. The combination of a) options that represent a range of identities, b) the ability to select multiple options, and c) a response that allows for respondents to self-identify, can be collectively used to create demographic question options that allow respondents to accurately convey their identities in ways that are more genuine and still useful for data analysis. Allowing respondents to select multiple options within a single survey question, as 78% of our respondents with queer genders and 9% of our respondents with a queer sexual, romantic, and related orientation did, creates a greater breadth of identity options, as it allows respondents to more clearly express their identities. While selecting multiple options provides for a richer dataset, it does sometimes require researchers to be careful when collating data to make sure they are selecting the correct subset of respondents. For example, in our dataset, when calculating the percent of participants who were likely cisgender women, we made sure we included only those who chose “female/feminine,” and not those who picked that option as well as other gender identities, such as “genderqueer.”

In discussing demographic questions, it is also important to discuss when and where demographic questions should be asked. Demographic questions should only be included in a survey if they are used to inform data analysis, not just simply out of habit, and their location in the survey (i.e. the beginning or the end) should be considered carefully [18]. The potential impact of identity on the experience you are trying to capture is another factor to consider in survey placement, as identity frequently influences how individuals experience a given situation. While demographic questions might seem to be good ‘warm-up’ questions for the beginning of a survey, for people with marginalized identities of any kind, demographic questions can be challenging and high risk. If queered demographic questions are at the end of a survey, they cannot influence how survey takers perceive the rest of the survey. Yet, placing demographic questions at the beginning of a survey may still be important, as it potentially helps identify if there are demographic patterns in those who do not complete a survey [55]. While there is little research on the best place to locate survey questions, empirical evidence is inconsistent regarding optimal placement, indicating that the context of the survey and the questions asked influence best practices for demographic question placement [18].

### Implementation challenges

Creating space for people with queer-spectrum identities is first and foremost an equity and social justice issue, which requires shifting the current cisheteronormative master narrative to a narrative that is queer-spectrum inclusive. Because cisteronormativity is deeply embedded in our society, those working to shift towards queer-inclusive demographic questions may experience barriers, even though queer-inclusive demographics questions do not decrease response rates from cishetero-respondents, and are supported by those with queer-spectrum identities [15, 21].

Given the persistent stigmas surrounding queer-spectrum identities in some social spaces, some may question the appropriateness of survey or interview questions that engage with queer identities or be concerned about how non-queer-spectrum people may perceive this inclusion. And, as exemplified by a small number of sarcastic and hostile answers on all of our survey types and similar findings of other researchers, a small subset of respondents will likely be upset by queer inclusive survey options [37]. We think of these responses not as a reason to discontinue inclusive practices, but rather an example of the importance of furthering inclusion of queer-spectrum identities.

A few large-scale surveys in the US, including the annual Freshman Survey and College Senior Survey (both administered by the Cooperative Institutional Research Program) and the NSFG now include some level of queer-inclusive demographic questions [15, 38]. The 2021 Canadian census is also integrating queer-inclusive questions [77]. And, research around queer-inclusive questions has shown that these questions do not decrease response rates of participants overall, which is also in line with our findings [15]. Even though queer-identity inclusion in surveys is not overwhelmingly desired by individuals who are both cisgender and heterosexual and queer-spectrum identity inclusion is less popular among people with conservative views [21], it is vital that queer-inclusion is not derailed into an issue of preference or popular opinion; it is a social justice and equity issue [78]. Just like many other issues of equity, it is vital to keep equity and social justice as central to the discussion, rather than focusing on giving space to discriminatory voices [62].

### Limitations

The need for a queer-spectrum demographic survey that is based on broad-scale research that explicitly elicits feedback from a range of queer-spectrum individuals is one of the clear outcomes of our study. We developed our survey through talking to a small number of queer-spectrum and cis-hetero individuals who the first author knew, as we were not satisfied with the existing surveys we found in the literature; a systematic survey of a much larger number of people is necessary to refine the survey questions. Additionally, our current data are only from undergraduate engineering and computer science students at four institutions of higher education, which is clearly not a representative sample of people in STEM or people more broadly; and, the open-ended survey was only implemented at one of the four institutions. Additionally, in our survey questions and interpretation, we have focused on identities individually, rather than approaching interpretation from an intersectional perspective. It is important for future studies to consider intersectional identities as queer-spectrum identities are culturally specific and fluid and because societal power structures that create inequities interact differently across multiple marginalized identities [35]. Future research needs to account for how different identities interact and include people with a range of identities to more broadly capture the range of queer-spectrum identities people hold.

### Counting is not enough–Future work

While developing strategies that effectively count queer-spectrum people in STEM (and beyond) is a first step, it is not an end in and of itself. Simply knowing that queer-spectrum people are under-represented but exist in STEM does not tell us about the specific problems and exclusionary systems that queer-spectrum people experience. It is vital to research queer-spectrum students’ experiences to address underlying systems of oppression, as well as interventions that work to create positive change. In the research realm there are several clear steps for moving forward: 1) further developing research-based queer-spectrum demographic survey questions, 2) conducting research on the problems that queer-spectrum individuals face in STEM (and beyond), and 3) developing practices to address existing problems.

The survey questions we developed and used in this study gives a starting point in developing questions that are based on larger-scale research. Just as the larger research community keeps refining language around race and ethnicity demographic questions through large-scale research [73], the same is needed for demographic questions related to queer-spectrum identities. Based on student responses from the study presented here, along with feedback from colleagues, we have developed a revised version of our survey (Box 2). However, these revised questions are still a stepping-stone to a more refined survey. Future research that includes both qualitative interview research and large-scale testing of the survey is necessary to create a well-developed, research-informed demographic survey.

Once there is a solid set of queer-inclusive demographics questions, and including them in surveys becomes common, it will be possible to more systematically learn about and develop interventions to address the problems that queer-spectrum scientists and students experience in STEM and elsewhere. The little research that currently exists shows that queer-spectrum people in STEM fields face particularly widespread systemic bias and discrimination, yet these issues are rarely discussed or addressed beyond queer-focused research [1, 10–13, 15, 16, 79]. While more research is needed, there are also clear existing problems that can be addressed now. Institutions need to make clearer statements and policies that actively address and counter discrimination, and inclusivity, equity, and social justice needs to be built into curriculum for students. As a whole, diversity, equity, inclusion, and social justice cannot be seen as a “niche” problem for people with specific identities: instead, they need to be addressed as a society-wide challenge.

## Supporting information

S1 Table Table of institutionally collected undergraduate student demographic information by semester and institution. We do not specify department or college to maintain confidentiality, however these data are specific to the college or department where we collected data at each institution.
(DOCX)

S2 Table An explanation of how our data address Engel and Schutt’s six questions to ask before using secondary data, as discussed in DeCarlo [63]. (DOCX)

S1 Appendix Explanation and suggestions for implementing queered survey questions. (DOCX)
