# Supplementary material for: Revealing the queer-spectrum in STEM through robust demographic data collection in undergraduate engineering and computer science courses at four institutions
Source: PLoS One. 2022 Mar 10;17(3):e0264267. doi: 10.1371/journal.pone.0264267 (PMC8912177; doi:10.1371/journal.pone.0264267)
Supplement: S1 Table — We do not specify department or college to maintain confidentiality, however these data are specific to the college or department where we collected data at each institution. (DOCX) [file pone.0264267.s001.docx]

**S1 Table: Table of institutionally collected undergraduate student demographic information by semester and institution.** We do not specify department or college to maintain confidentiality, however these data are specific to the college or department where we collected data at each institution.

| **Mid-Atlantic Public** | | |  |  |  |  |  |  |  |  |  |  |
| --- | --- | --- | --- | --- | --- | --- | --- | --- | --- | --- | --- | --- |
| Total students |  | Male | Female | Nonresident Alien | American Indian/ Alaskan Native | Asian | Black | Hispanic | Native Hawaiian/ Pacific Islander | Two or More Races | Unknown | White |
| 4,111 | Fall 17 | 83% | 17% | 19% | 0% | 2% | 2% | 3% | 0% | 3% | 0% | 71% |
| 3,811 | Fall 18 | 82% | 18% | 19% | 0% | 2% | 2% | 3% | 0% | 2% | 0% | 71% |
| 3,641 | Fall 19 | 81% | 19% | 15% | 0% | 2% | 2% | 3% | 0% | 3% | 1% | 74% |
|  |  |  |  |  |  |  |  |  |  |  |  |  |
| **Rocky Mountain Private** | | | |  |  |  |  |  |  |  |  |  |
| Total students |  | Male | Female | Nonresident Alien | American Indian/ Alaskan Native | Asian | Black | Hispanic | Native Hawaiian/ Pacific Islander | Two or More Races | Unknown | White |
| 596 | Fall 17 | 78% | 22% | 11% | 0% | 6% | 2% | 14% | 0% | 5% | 1% | 60% |
| 608 | Fall 18 | 77% | 23% | 10% | 0% | 6% | 2% | 14% | 0% | 6% | 2% | 61% |
| 576 | Fall 19 | 76% | 24% | 10% | 0% | 6% | 3% | 11% | 0% | 6% | 1% | 61% |
|  |  |  |  |  |  |  |  |  |  |  |  |  |
| **Rocky Mountain Public** | | | |  |  |  |  |  |  |  |  |  |
| Total students |  | Male | Female | Nonresident Alien | American Indian/ Alaskan Native | Asian | Black | Hispanic | Native Hawaiian/ Pacific Islander | Two or More Races | Unknown | White |
| 2,654 | Fall 17 | 76% | 24% | 8% | 0% | 2% | 1% | 10% | 0% | 3% | 2% | 73% |
| 2,683 | Fall 18 | 75% | 25% | 7% | 0% | 2% | 1% | 11% | 0% | 4% | 1% | 72% |
| 2,602 | Fall 19 | 74% | 26% | 6% | 0% | 2% | 1% | 12% | 0% | 5% | 1% | 72% |
|  |  |  |  |  |  |  |  |  |  |  |  |  |
| **Rocky Mountain Public Teaching** | | | | |  |  |  |  |  |  |  |  |
| Total students |  | Male | Female | Nonresident Alien | American Indian/ Alaskan Native | Asian | Black | Hispanic | Native Hawaiian/ Pacific Islander | Two or More Races | Unknown | White |
| 608 | Fall 19 | 86% | 14% | Unknown | 0% | 9% | 9% | 26% | 0% | Unk. | Unknown | 47% |
