# Supplementary material for: Revealing the queer-spectrum in STEM through robust demographic data collection in undergraduate engineering and computer science courses at four institutions
Source: PLoS One. 2022 Mar 10;17(3):e0264267. doi: 10.1371/journal.pone.0264267 (PMC8912177; doi:10.1371/journal.pone.0264267)
Supplement: S2 Table — (DOCX) [file pone.0264267.s002.docx]

S2 Table:

An explanation of how our data address Engel and Schutt’s six questions to ask before using secondary data, as discussed in DeCarlo [58].

| Question to address when using secondary data (Engel and Schutt (2016) as discussed in DeCarlo [58] | How our data addresses this question |
| --- | --- |
| 1. What were the researcher’s goals in collecting the data? | The researchers sought to accurately capture students’ gender identity, allowing the researchers to use it in analyses of students’ experiences, such as a mediating or moderating variable in statistical analyses. Therefore, our goal of capturing how students responded to the gender questions is consistent with the original goals. |
| 2. What data were collected, and what were they intended to measure? | Data on students’ genders and sexual, romantic, and related orientations were collected in a range of ways, with the intent of capturing students’ identities. |
| 3. When was the information collected? | The information was collected from Fall 2017-Spring 2020. This timeframe is both recent, and therefore relevant in answering our research questions, and was collected over a span of 3 academic years. Therefore, the timeline of data collection is short and continuous. Additionally, the Spring 2020 data were collected at the beginning of the semester, prior to COVID-19 becoming a global pandemic and disrupting students’ daily and academic lives. |
| 4. What methods were used for data collection? Who was responsible for data collection, and what were their qualifications? Are they available to answer questions about the data? | The data were collected using a Qualtrics survey by Ph.D. level researchers with degrees in educational research and with 8+ years of related research experience each. Two of the authors on this paper are part of the data collection team and work closely with the others involved in data collection. Thus, there are no barriers to obtaining information about the data. |
| 5. How is the information organized? Are there identifiers used to identify different types of data available? | The information is organized in spreadsheets that contain the output provided by Qualtrics. There are identifiers available to identify the different types of data available. |
| 6. What is known about the success of the data collection effort? How are missing data indicated and treated? What kind of documentation is available? How consistent are the data with data available from other sources? | As discussed in the “course contexts” section of the methods, the overall percent of students who both completed the survey and consented to have their data included in our study was 70-80% within a given course. This response rate is well above the median response rate in education research of 50% [52]. All students who responded to the survey are in the dataset we used for this study and we explicitly include the number of students who left a question blank in our results. The approach towards missing data in the study and data and documentation available are consistent across all of our data sources, as they were all collected by the same research team. |
