# Supplementary material for: Revealing the queer-spectrum in STEM through robust demographic data collection in undergraduate engineering and computer science courses at four institutions
Source: PLoS One. 2022 Mar 10;17(3):e0264267. doi: 10.1371/journal.pone.0264267 (PMC8912177; doi:10.1371/journal.pone.0264267)
Supplement: S1 Appendix — (DOCX) [file pone.0264267.s003.docx]

**S1 Appendix: Explanation and suggestions for implementing queered survey questions**

Below are the revised queered demographics questions as of Fall 2021, developed by Dr. Aramati Casper. While these questions reflect feedback from both in-classroom use and consultations with both queer and cis-hetero individuals, they are still in development. Additionally, language use around queer identities varies in many different ways. Depending on the population you are working with you may need to make revisions to fit your specific population. In revising, please make sure to ask the question: whose interests am I centering, those with marginalized queer identities, or those with dominant identities?

In developing this survey, I have worked to maintain centering the interests of queer individuals over other concerns. For example, there are options that arguably conflate gender and sex. The “binary gender” categories (e.g. Female or Feminine or Woman) were constructed with a lot of thought and discussion to convey their inclusive nature. While “transman” and “transwoman” are generally considered to be outdated language, in some geographical areas they are still used, as transgender students have stated that they read “man” and “woman” to have an implicit cisgender in front of them. Additionally, some queer individuals have expressed that they affiliate with female as a gender, even though in the academic literature female is usually considered a sex, and woman the associated gender. Similarly, intersex is a sex, but some intersex individuals also use this as their gender. Navigating these language challenges as effectively as possible is one the goals of our future research.

We also include suggested text for the beginning of the demographics section, which provides a rationale for asking these questions. While you may wish to modify this text, providing some rationale helps participants understand why we want this information.

If you have any questions, are curious if there is a further updated survey, or are potentially interested in collaborating on further research using and refining these questions, please contact Dr. Aramati Casper at [aramaticasper@gmail.com](mailto:aramaticasper@gmail.com).

**Recommended text before the entire demographic question section**:

This section asks a series of demographics questions. While everyone has their own unique experience, your answers to these questions will help us identify patterns in these experiences that may be linked to identity.

**For both questions, make sure the question is set up such that respondents can select any number of choices, including mixing “self-identify” with pre-written answers.**

Please indicate the identity(ies) you feel most closely describe your

current gender(s). Select all that apply.

1) Agender

2) Female, Feminine, or Woman

3) Genderfluid

4) Genderqueer or Non-binary

5) Gender non-conforming

6) Intersex

7) Male, Masculine, or Man

8) Not cisgender, but I don’t identify with a specific identity

9) Questioning or Figuring it out

10) Transgender

11) Two-spirit or other Traditional or Indigenous genders

12) Prefer not to respond

13) I don’t understand the question

14) Prefer to self-identify ___________________

Please indicate the identity(ies) you feel most closely describe you current sexual, romantic, or related orientation(s). Select all that apply.

1) Asexual or Ace spectrum

2) Bisexual

3) Gay

4) Lesbian

5) Not heterosexual, but don’t identify with a specific identity

6) Pansexual or Omnisexual

7) Questioning or Figuring it out

8) Straight or Heterosexual

9) Queer

10) Prefer not to respond

11) I don’t understand the question

12) Prefer to self-identify __________________

**If you are trying to customize these questions you may also want to ask:**

Is there a way your identity could be better represented in the gender identity survey question? If yes, please describe:

Is there a way your identity could be better represented in the sexual, romantic, or related orientation survey question? If yes, please describe:
